# Past and Future Work

**Published:** 1921-09-24

**Authors:** 


### THE ROCKEFELLER FOUNDATION.

### Past and Future Work.

It is the aim of the Rockefeller Foundation to contribute to the progress of the world by helping to increase the common store of knowledge of the causes of disease, and through demonstrations and the services of experts to diffuse this information as widely as possible among all the peoples of the world. The English public will remember the munificent provision of the Foundation in connection with University College Hospital, which provided a large sum to be divided between buildings and endowment for increased educational and research activities. The recently published report on the work of the Foundation shows that this gift is but an example of wide-spread work on the same lines in many lands.

The funds and property of the Rockefeller Foundation were at December 31, 1920, valued at $171,000,000, and the expenditure for the year ending on that date was over seven million dollars. The record of work included the aiding of six medical schools in Canada, the gift to London mentioned above, a million francs for research in Belgium, the construction and maintenance of a medical school in Peking, and the aiding of thirty-one hospitals in China, support of work on hygiene and public health at Johns Hopkins University and in Brazil, help to a campaign against yellow fever in Africa and South America, work on malaria, hook-worm, and other tropical diseases, a gift of a million dollars to the relief fund for European children, and help to other objects within the scope of the Foundation.

Prevention is the watchword. It is pointed out that a railway spends more money on train and track inspection than on wreck crews, and that the average automobile owner is on the watch for signs of motor trouble and does not wait until there is a breakdown. But the executive of the Foundation recognise that their work is far from being simple. Public authorities can control wholly or in part only about 20 per cent. of the diseases by which people are crippled or killed, typhoid, scarlet fever, and malaria can be either entirely prevented or kept from spreading; but tuberculosis, measles, diphtheria, pneumonia, influenza, and many other maladies are either less perfectly understood or do not respond so readily to control efforts. Valuable as this community-protection against contagious diseases is, it is suggested that about 80 per cent. of the menace to life is not dealt with, at any rate directly, by public authorities. The idea of prevention must, therefore, have limited influence until it is accepted not merely as a Government policy, but as a guiding principle in individual lives. Large though the resources of the Rockefeller Trust are, in such a vast field they must be cautiously administered, and the chief line taken, so far, is the vital and fundamental necessity of the training of doctors. The executive consider that progress cannot better be helped than by helping strategically placed medical schools in various parts of the world to increase their resources and to improve their teaching and research.

As the work of the Foundation became known applications for assistance began to pour in, but as the work was restricted almost exclusively to a few broad undertakings only a few could be responded to; 788 requests were received and 50 were granted—124 applications were from local churches and institutions, 114 in connection with investigation, reward, or purchase of alleged medical discoveries, and 115 of a personal nature—all of these had to be declined.

The report is reticent on the subject of any fresh developments, and the announced programme of work for the current year is almost entirely in connection with the support of work already launched or aided.

